# Molecular Crossroads: Shared and Divergent Molecular Signatures in Alzheimer’s Disease and Dementia with Lewy Bodies

**DOI:** 10.3390/ijms262411811

**Published:** 2025-12-07

**Authors:** Sandesh Neupane, Tibor Hortobágyi

**Affiliations:** 1 Institute of Neuropathology, University Hospital of Zurich, University of Zurich, Schmelzbergstrasse 12, 8091 Zurich, Switzerland; 2 Department of Neurology, Faculty of Medicine, University of Debrecen, 4032 Debrecen, Hungary

**Keywords:** Alzheimer’s disease, dementia with Lewy bodies, amyloid-β, tau, alpha-synuclein, biomarkers

## Abstract

Alzheimer’s disease (AD) and dementia with Lewy bodies (DLB) are the two most common forms of dementia due to neurodegeneration. AD is characterized by extracellular amyloid-β (Aβ) plaques and intracellular tau neurofibrillary tangles, whereas DLB is defined by α-synuclein (α-Syn)-containing Lewy bodies. Although AD and DLB exhibit divergent core features, the disorders frequently co-occur and converge on shared endpoints. Co-pathology is common and linked to more severe cognitive decline, faster progression, and clinicopathological heterogeneity. Here, we discuss the current understanding of shared and unique clinical and neuropathological features of AD and DLB. We compare genetic risk and pathological drivers (Aβ and tau in AD; α-Syn in DLB) and their overlapping co-pathology, and review downstream mechanisms—mitochondrial dysfunction, oxidative stress, neuroinflammation, and cerebrovascular contributions, including cerebral amyloid angiopathy. We highlight recent findings from state-of-the-art multi-omics (transcriptomic, proteomic, metabolomic, and single-cell/spatial studies) that reveal convergent and disease-specific molecular signatures of AD and DLB. We outline a framework for emerging next-generation biomarkers—from blood-based and cerebrospinal fluid assays to imaging and digital measures—for diagnosis and stratification, and discuss potential translational implications. Together, these advances help to disentangle shared from disease-specific mechanisms, which is essential for improved diagnosis and the development of precise, disease-modifying therapies.

## 1. Introduction

On 3 November 1906, at a German psychiatrists meeting in Tübingen, Germany, Alois Alzheimer, a psychiatrist and neuropathologist gave a talk on “A peculiar severe disease process of the cerebral cortex”, describing a woman named Auguste Deter with symptoms of presenile dementia, and reported the post-mortem autopsy of her brain that showed senile plaques and neurofibrillary tangles [1,2]. A few years later in 1910, Emil Kraepelin, a German psychiatrist, named the disease condition “Alzheimer’s disease” [3,4]. Alzheimer’s disease (AD) is the most common progressive neurodegenerative disorder caused by neuronal cell death. AD currently affects around 50 million patients globally and this number is projected to reach approximately 152 million by 2050 [5,6]. According to global burden-of-disease estimates, AD and other dementias accounted for 25.3 million disability-adjusted life years (DALYs) worldwide in 2019, increased from 9.66 million DALYs in 1990, reflecting a substantial rise over this period [7,8,9]. AD is prevalent among adults aged 65 years or older, and has a life expectancy of about 3–10 years [10]. Depending on the stage, AD patients often exhibit a severe decline in cognitive and behavioural ability, initially with memory and spatial navigation problems, and other symptoms such as executive dysfunction (planning/organization), impaired reasoning and judgement, attention and concentration problems, personality and behaviour changes, mood changes, social withdrawal, agitation and aggression, paranoia/delusions, and sleep disturbances [11,12,13,14].

Early stage AD exhibits histological alterations and atrophy in the entorhinal cortex within the hippocampus of the medial temporal lobe, a brain region crucial for episodic memory formation and consolidation and spatial navigation [15,16,17]. Biochemists George Glenner and Caine Wong reported the accumulation of a 4 kDa peptide, which they termed “amyloid-β protein” (Aβ), the major constituent of extracellular amyloid plaques [18,19]. Similarly, Inge Grundke-Iqbal and Khalid Iqbal identified microtubule-associated protein tau (MAPT) in some neurofibrillary tangles and plaque neurites in the AD brain [20,21].

Dementia with Lewy bodies (DLB) is an age-associated neurodegenerative disorder that is less common than AD [22]. DLB is a member of a group of neurodegenerative diseases referred to as synucleinopathies, which also include Parkinson’s disease (PD) and multiple system atrophy (MSA). Synucleinopathies are a group of disorders characterized by pathological accumulation of the misfolded form of the small presynaptic protein alpha-synuclein (α-Syn), which is encoded by the gene SNCA. Major synucleinopathies are Lewy body diseases (LBDs) [23]. LBDs refer to a group of disorders comprising Parkinson’s disease (PD), Parkinson’s disease dementia (PDD), and dementia with Lewy bodies (DLB), which share the neuropathological hallmark of deposition of Lewy bodies within neurons and extensive axonal Lewy neurites [24,25]. However, among synucleinopathies, MSA is characterized by accumulation of inclusions known as glial cytoplasmic inclusions containing synuclein aggregates in oligodendrocytes, while Lewy body pathology is absent and, therefore, the LBD group does not include MSA [24,26,27,28].

DLB has an incidence of 0.5–1.6 per 1000 person-years and affects an estimated 1.4 million people in the United States [29]. It accounts for ~7.5% of all dementia cases [30]. DLB typically begins in the late fifties and constantly increases with age [31,32], and it is more prevalent in males than females [33]. Historically, in 1912, Jakob Heinrich Lewy, who was studying Parkinson’s disease at Alois Alzheimer’s laboratory, observed eosinophilic intraneuronal inclusion bodies, which were named Lewy bodies by Nikolaevich Tretiakoff in 1919 [34]. DLB gained momentum in 1961, when Okazaki et al. reported that patients with dementia who died shortly thereafter and had severe extrapyramidal rigidity [35]. Later, Japanese psychiatrist Kenji Kosaka and colleagues observed Lewy bodies in the brainstem and cerebral cortex in post-mortem autopsy samples from more than 20 patients with different types of cognitive impairment [36,37]. In 1995, the term “dementia with Lewy bodies” was first proposed at the First International Workshop (Newcastle upon Tyne, England), with the diagnostic criteria focused on three core features: impairment in cognitive function, hallucinations, and Parkinsonian features [36,38,39]. In 1997, Spillantini and colleagues identified α-Syn as a major component of Lewy bodies in the brain tissue from patients with DLB and PD [40].

AD and DLB frequently converge clinically and pathologically, complicating diagnosis, prognostication, medication selection, and eligibility for clinical trials. In this review, we briefly compare the clinical and neuropathological features of AD and DLB. We integrate evidence across fluid biomarkers, genetics, and imaging to delineate shared versus disease-specific biology. Finally, we translate the evidence from the findings into therapeutic implications and outline new future research directions with potential next steps for targeted treatments.

## 2. Clinical and Neuropathological Overlap Between AD and DLB

### 2.1. Clinical Features of AD

AD usually co-exists with ageing and presents with cognitive, behavioural, and psychological symptoms. In the early stage, individuals with AD show amnestic features such as memory loss, trouble learning new information, repeating questions, misplacing items, and difficulty with spatial navigation [14,41]. As the disease progresses, the moderate stage includes memory loss; disorientation of time and place; language difficulties; and impaired executive function such as poor reasoning and planning, with changes in mood (apathy, irritability, or depression), difficulty in solving complex daily tasks, and impaired judgement. In the late stages, AD patients show global cognitive failure, dependency for activities of daily living, gait impairment, dysphagia, and other complex neurological and psychiatric symptoms such as agitation, delusions or occasional hallucinations, and extrapyramidal symptoms such as rigidity or gait disturbance [14,42]. Initially, in 1984, the criteria were set by the National Institute of Neurological and Communicative Disorders and Stroke and the Alzheimer’s Disease and Related Disorders Association (NINCDS–ADRDA criteria for AD) as a suitable procedure for the clinical diagnosis of AD and have recently been updated [43,44,45]. The National Institute on Aging and Alzheimer’s Association (NIA–AA) updated the clinical approach guidelines, introduced the use of biomarkers, and proposed a new biological scheme: amyloid, tau, neurodegeneration (A/T(N)) for research [46]. In day-to-day practice, clinicians still diagnose the disease based on medical records, physical and neurological examinations, neuropsychological evaluation, and neuroimaging [47].

### 2.2. Clinical Features of DLB

Individuals with DLB also show cognitive impairment with characteristic non-amnestic features that often different from those of typical AD. The major symptoms of DLB are progressive cognitive or visuospatial impairment with fluctuating attention and alertness, parkinsonism (rigidity, slowness, tremor, shuffling gait, and falls), recurrent visual hallucinations, REM sleep behaviour disorder (RBD), and antipsychotic sensitivity causing worse parkinsonism, confusion, and sleepiness [48,49]. In the later stages, DLB patients commonly develop autonomic instability (orthostatic hypotension and urinary incontinence), repeated falls, and increased sensitivity to antipsychotics [33]. The 2017 DLB Consortium criteria diagnose probable DLB if there are two or more core clinical features, or one core feature plus an indicative biomarker (reduced striatal dopamine-transporter uptake (positron emission tomography/single-photon emission computed tomography, PET/SPECT), reduced myocardial iodine-123-metaiodobenzylguanidine (MIBG) uptake, or polysomnographic confirmation of rapid eye movement (REM) sleep without atonia) [22,50,51]. Many symptoms of DLB overlap with those of PD; hence, the timing of the symptoms is diagnostically crucial. According to the “1-year rule”, dementia that begins before or within 1 year of the onset of parkinsonism helps separate DLB from PDD. DLB is favoured when dementia precedes or appears within 1 year of the onset of parkinsonism [38,50,52,53].

AD and DLB have some distinct early symptoms that can help differentiate them. AD starts with prominent episodic memory loss, a steady decline, and spatial navigation deficits, and features like hallucinations or parkinsonism usually appear late [47,54]. However, in DLB, early visual hallucinations, fluctuating cognitive features, RBD, and parkinsonism are hallmarks; autonomic failure and marked antipsychotic sensitivity increase the diagnostic confidence for DLB [55,56]. On bedside testing, DLB shows visuospatial and attentional deficits disproportionate to memory impairment, whereas AD is characterized by predominant amnestic deficits. The timing of motor signs is important: parkinsonism near or preceding cognitive symptoms supports DLB, while late parkinsonism in a prolonged amnestic course favours AD with superimposed extrapyramidal signs [57]. Recognition of these discriminators reduces misdiagnosis and avoids toxic antipsychotic exposure in DLB. Overall, although almost the same neurological and psychiatric symptoms may occur in AD and DLB, there are differences in time of onset, prevalence, and severity during the disease course [58,59]. Cases with multiple co-pathologies are associated with increased odds of late-onset hallucinations [59]. Memory loss is the most prevalent early sign in AD, whereas in DLB, other cognitive symptoms (planning, judgement, and problem solving) prevail as the presenting sign. Movement disorders, hallucinations, sleep disorders, fluctuations in alertness and attention, and autonomic nervous system impairment signs (orthostatic hypotension, urinary incontinence) are more frequent and have an earlier onset in DLB than in AD.

### 2.3. Neuropathological Features of AD

At the autopsy, AD brains often show moderate cerebral cortical atrophy primarily with narrowed gyri and widened sulci, early and significant atrophy of the medial temporal lobe (hippocampal/entorhinal atrophy), and symmetrical dilation of the lateral ventricles (hydrocephalus ex vacuo) compared with healthy age-matched controls [60,61,62]. Microscopically, AD brains show accumulation of extracellular Aβ (diffuse and neuritic) and intraneuronal neurofibrillary tangles composed of hyperphosphorylated tau [63,64,65]. In a landmark study by Braak H. and Braak E., tau pathology progresses in a predictable Braak sequence: transentorhinal (Stage I–Stage II) → limbic/hippocampal (Stage III–Stage IV) → widespread neocortical (Stage V–Stage VI), tracking clinical severity; Aβ deposition follows Thal phases from neocortex to deeper structures [66,67,68,69]. Thal phases (1–5) stage the spread of amyloid-β plaques in AD: starting in the neocortex (1), then hippocampal/allocortical regions (2), striatum and diencephalon (3), brainstem (4), and finally the cerebellum (5) [69]. Neuroinflammatory changes such as activated microglia and astrocytosis, together with synaptic loss and neuronal death, accompany these lesions, and tangle burden correlates with cognitive impairment [60]. Current NIA–AA neuropathologic guidelines report “AD neuropathologic change” on a continuum, integrating Braak neurofibrillary tangle stage, Thal Aβ phase, and neuritic plaque scores [70].

### 2.4. Neuropathological Features of DLB

DLB brains also show cortical atrophy, but it is less severe than in AD [24]. Most prominently in early stages, for instance in the medial temporal lobe (hippocampus), atrophy is more profound in AD patients than in DLB. DLB patients show Lewy pathology, which is observed in the brainstem (mainly the substantia nigra), limbic, and neocortical regions [71]. DLB brains mainly exhibit depigmentation of the substantia nigra and locus coeruleus due to loss of the majority of pigmented neurons. In later stages, DLB individuals’ brains show moderate cortical atrophy [24,72,73,74]. Microscopically, DLB patients show two general types of Lewy bodies: classic brainstem Lewy bodies with a dense eosinophilic core and a pale halo (visible by haematoxylin and eosin staining), and cortical Lewy bodies, which are smaller, lack the halo, and are mainly observed by α-Syn immunohistochemistry. α-Syn is the primary component of Lewy bodies [25,75,76]. However, post-mortem studies of DLB brains suggest that microglial activation is not prominent in DLB compared to AD [77].

Comparatively, DLB post-mortem tissue shows widespread cortical and limbic α-Syn-containing inclusions, whereas AD shows medial temporal (hippocampal) atrophy with abundant neuritic Aβ plaques and high-stage tau tangles.

Co-pathology is common, but lesion type, regional burden, and staging (Braak/Thal) usually distinguish the two [24,78,79,80]. Pure phenotypes have exclusively either AD or DLB pathology, whereas mixed phenotypes present with both based on the standard neuropathological examination protocols. The high proportion of mixed AD + DLB cases and prevalence of other co-pathologies negatively influence the accuracy of clinical diagnosis versus neuropathology, with implications for clinical trial enrolment and evaluation [81].

**Figure 1:**
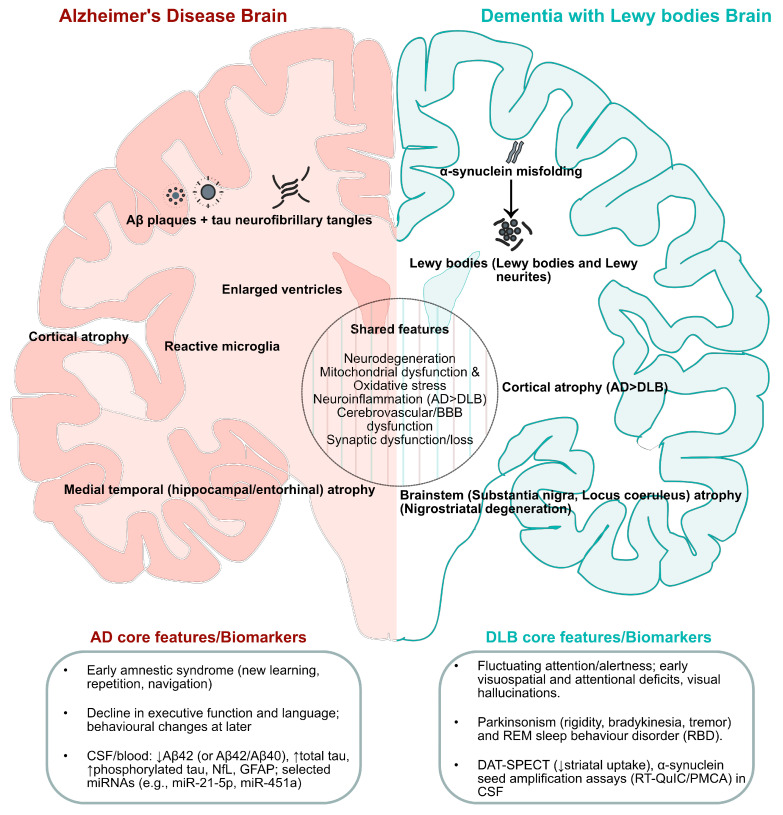
Comparison of shared and discriminating features of AD and DLB. Left hemisphere (AD-predominant): extracellular Aβ plaques and intraneuronal tau tangles accompanied by medial–temporal and cortical atrophy and ventriculomegaly. Right hemisphere (DLB-predominant): α-synuclein Lewy bodies and neurites in brainstem (substantia nigra, locus coeruleus). The middle circular band summarizes the shared features: mitochondrial dysfunction/oxidative stress, neuroinflammation, cerebrovascular/BBB, and synaptic/proteostasis–lysosome–autophagy stress. The bottom boxes summarize the core clinical profiles and indicative biomarkers for AD and DLB. AD, Alzheimer’s disease; DLB, dementia with Lewy bodies; Aβ, amyloid-β; α-Syn, α-synuclein; DAT, dopamine transporter; GFAP, glial fibrillary acidic protein; LBV, Lewy body variant of AD; NfL, neurofilament light; RT-QuIC, real-time quaking-induced conversion; PMCA, Protein Misfolding Cyclic Amplification; DAT-SPECT, dopamine transporter single-photon emission computed tomography. Downward (↓) arrows indicate decreases in protein levels or uptake.

An overview of the shared versus disease-specific features is shown in Figure 1.

## 3. Shared and Divergent Molecular Pathways

### 3.1. From Etiology to Genetics: AD Versus DLB

The exact cause of AD remains a mystery to researchers and clinicians; however, ageing is the most predominant risk factor. Various genetic and epigenetic factors, together with environmental factors including lifestyle, diet, chronic stress, infections, hearing loss, hypertension, alcohol, air pollution, midlife cholesterol levels, vision loss, neuroendocrine changes, and other lifelong chemical exposures, can disrupt immune homeostasis, triggering neurodegenerative cascades [82,83,84,85].

Pathogenic variants in APP (amyloid precursor protein), PSEN1 (presenilin-1), PSEN2 (presenilin-2) are rare and cause early-onset familial AD, and the ε4 and ε2 variants of APOE (apolipoprotein E) are AD susceptibility alleles. However, despite the many known genetic associations, AD still lacks confirmed modifiable risk factors or preventive therapies [86]. Phenomic and genomic studies such as GWASs (genome-wide association studies), PheWASs (phenome-wide association studies), and Mendelian randomization analyses of the UK Biobank dataset reported 75 loci (42 novel) linked to pathways enriched for amyloid/tau, lipid metabolism, endocytosis, and immunity [87,88,89].

Multiple hypotheses have been proposed to describe the complex etiology of AD. These include the amyloid cascade hypothesis, tau propagation (prion-like spread of tau), the cholinergic deficit hypothesis, the mitochondrial cascade hypothesis, calcium homeostasis dysregulation, neurovascular dysfunction, chronic neuroinflammation, dysregulation of metal ion homeostasis, and impaired glymphatic clearance of proteins in the brain [87,90,91,92]. Each model captures a different dimension of the pathology, suggesting that AD likely results from a convergence of multiple factors rather than a single cause.

The exact cause of DLB also remains unclear. Multiple lines of evidence support a multifactorial cause which includes ageing, genetic vulnerability and environmental factors as in AD [93,94]. Several environmental and lifestyle factors increase the risk of cognitive impairment and dementia. A meta-analysis by Zhao et al. reported that air pollution, tobacco smoke, and pesticides could elevate the risk of dementia, whereas exposure to electromagnetic fields was positively associated with it [95]. Solvents and aluminum were found to be hazards for dementia, and living in rural areas and more deprived neighbourhoods were associated with higher dementia risk. These links are largely observational, correlational, and inconsistent across studies, so causality has not yet been clearly established. Regarding dementia types, it has been shown that air pollution is associated with an increased risk of developing DLB [96].

In 2021, Chia et al. identified five genome-wide significant risk loci (GBA, BIN1, TMEM175, SNCA and APOE) using whole-genome sequencing on large DLB cohorts and neurologically healthy controls. Polygenic risk analyses supported shared genetic profiles of DLB with AD and PD [97]. Functionally and etiologically, these findings place DLB at the intersection of PD and AD co-pathology [98]. GBA, SNCA and TMEM175 are well-established PD risk loci [99], whereas APOE and BIN1 are known AD risk loci [100]. Similarly, Guerreiro and colleagues further complemented these results with an unbiased GWAS of DLB that used a two-stage design and identified five loci in discovery: APOE, BCL7C/STX1B, SNCA, GBA, and GABRB3. Replication confirmed the genome-wide significance of APOE, SNCA, and GBA, while GABRB3 did not retain significance in pathologically confirmed subsets. The study also provided evidence for a novel candidate locus: CNTN1 [101].

### 3.2. Pathogenic Frameworks: Aβ/Tau/Cholinergic Hypotheses in AD and α-Syn Pathology in DLB

In 1992, John Hardy and David Allsop put forth the well-known “Aβ Cascade Hypothesis”, which posits that the accumulation of misfolded Aβ peptides and their insufficient clearance in the brain initiates AD [102]. Aβ deposition is thought to set off a toxic cascade leading to synaptic dysfunction, tau tangle formation, and downstream widespread neurodegeneration [102,103,104]. Under normal physiological conditions, first, APP is cleaved by the brain’s major β-secretase, β-site APP cleaving enzyme 1 (BACE1), releasing the large soluble sAPPβ fragment and leaving behind a 99-amino-acid, membrane-tethered C-terminal fragment (CTF99). Second, the multisubunit γ-secretase complex cleaves this CTF99 within the membrane, producing Aβ peptides of various lengths. The two most common Aβ species are Aβ_40_ (40 amino acids) and Aβ_42_ (42 amino acids). Aβ_40_ is the more abundant form, but the slightly longer Aβ_42_ is prone to misfolding and aggregation into toxic fibrils [91,104,105,106,107].

The cholinergic signalling deficit in AD suggests that early degeneration of basal forebrain cholinergic neurons and cortical acetylcholine deficit correlates with cognitive impairment, explaining symptomatic benefit of acetylcholinesterase inhibitors; however, this hypothesis fails to explain the major neurodegeneration in AD [104,108,109].

In healthy neurons, MAPT-encoded tau supports different cellular functions and the transport of axonal nutrients. Tau is mainly involved in promoting the assembly and stabilization of neuronal microtubules [110]. In AD, tau is misfolded, hyperphosphorylated, and becomes prone to self-aggregation, forming insoluble straight filaments and paired helical filaments as neurofibrillary tangles [111,112]. These aggregated forms of tau disrupt neuronal function and contribute to neurodegeneration. Misfolded, hyperphosphorylated tau can seed and further spread in a prion-like manner [113,114]. Furthermore, numerous studies have demonstrated that tau pathology is also triggered by aggregated Aβ [114,115]. In a study by Bencze et al., post-mortem middle frontal gyrus and anterior hippocampus samples from AD patients were used to examine the regional relationship between Lemur Tyrosine Kinase 2 (LMTK2) and phospho-tau. They found that LMTK2 expression was significantly reduced in neurofibrillary tangle-affected regions and showed a strong inverse correlation with phospho-tau levels, indicating that decreased LMTK2 is associated with tau pathology rather than a general feature of AD brains [71]. Additionally, TAR DNA-binding protein 43 (TDP-43), a major hallmark of amyotrophic lateral sclerosis, was detected in human post-mortem brains from patients with AD and DLB; phosphorylated TDP-43 was present in most AD cases and in a subset of DLB cases [116].

**Figure 2:**
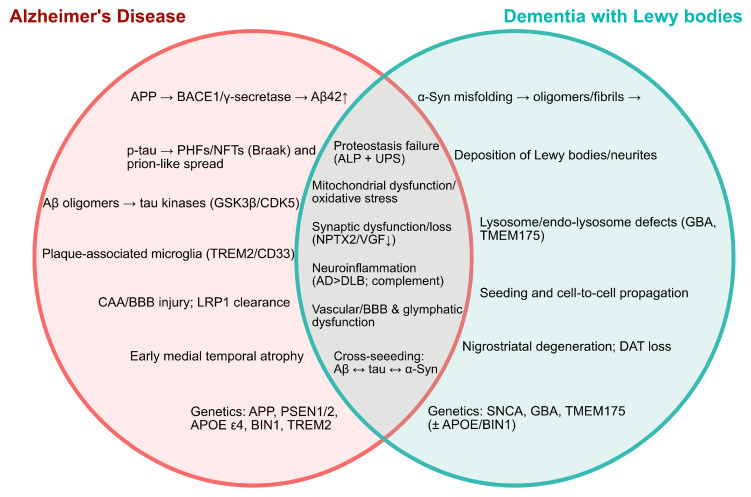
Venn diagram comparing the key unique and shared molecular features of AD and DLB. Left circle shows AD-specific drivers (Aβ plaques and tau neurofibrillary tangles). Right circle shows DLB-specific drivers (α-synuclein misfolding; Lewy bodies/Lewy neurites; and nigrostriatal degeneration). The overlap highlights shared cascades. α-Syn, α-synuclein; Aβ, amyloid-β; AD, Alzheimer’s disease; ALP, autophagy–lysosome pathway; APOE, apolipoprotein E; APP, amyloid precursor protein; BBB, blood–brain barrier; BACE1, β-site APP-cleaving enzyme-1; BIN1, bridging integrator 1; CD33, cluster of differentiation 33 (Siglec-3); CDK5, cyclin-dependent kinase 5; CAA, cerebral amyloid angiopathy; DAT, dopamine transporter; DLB, dementia with Lewy bodies; GBA, glucocerebrosidase (gene); GSK3β, glycogen synthase kinase-3β; LRP1, low-density lipoprotein receptor-related protein 1; NFTs, neurofibrillary tangles; NPTX2, neuronal pentraxin-2; PHFs, paired helical filaments; PSEN1, presenilin-1; PSEN2, presenilin-2; SNCA, α-synuclein (gene); TMEM175, transmembrane protein 175; TREM2, triggering receptor expressed on myeloid cells 2; UPS, ubiquitin–proteasome system; VGF, VGF nerve growth factor inducible; α-syn, alpha-synuclein; p-tau, phosphorylated tau, Single arrows (→), directional/pathological progression; double-headed arrows (↔), bidirectional cross-seeding interactions; and upward (↑) or downward (↓) arrows indicate relative increases or decreases in protein levels or pathway activity, respectively.

Intraneuronal accumulation of α-Syn inclusions is a prominent feature of DLB, with Lewy bodies in neuronal soma and Lewy neurites in processes [25,117]. Natively unfolded α-Syn misfolds into β-sheet-rich oligomers that build into protofibrils and finally insoluble fibrils, a stepwise process that is often accelerated at membranes. These pathogenic assemblies spread cell-to-cell when seeds are released from neighbouring or dying neurons (including via vesicles) and then taken up by neighbouring cells, where they act as templates for further aggregation and toxicity [118]. Importantly, α-Syn can adopt distinct “strains,” each with its own biochemical features and seeding potential [119,120]. In DLB, strain properties favour robust neuronal seeding and Lewy-type pathology, which helps to explain the distinct pathological signature of cortical and subcortical involvement [121]. A mechanism-focused Venn diagram highlighting the key unique and shared molecular features of AD and DLB is shown in Figure 2.

Co-pathology among Aβ, tau, and α-Syn is common across AD/DLB and associates with more severe phenotypes and faster cognitive decline. Guo et al. investigated whether α-Syn can directly cross-seed tau fibrillization and showed that distinct α-Syn “strains” differentially cross-seed tau in neuronal cultures and in vivo [122]. Bassil et al. showed that, in a mouse model injected with α-Syn preformed fibrils, Aβ plaques facilitate the seeding and network spread of α-Syn and promote the development of hyperphosphorylated tau pathology. This supports a feed-forward co-pathology loop, whereby Aβ plaques enhance endogenous α-Syn seeding and the spreading of pathology [123]. Another line of research by Zempel and colleagues showed that Aβ oligomers acutely drive somatodendritic mis-sorting of tau, accompanied by local dendritic spine loss and increased tau phosphorylation via Ca^2+^/CDK5-dependent pathways, thereby priming tau aggregation in primary rat hippocampal neurons [124]. Giasson et al. demonstrated that α-Syn directly seeds tau and that co-incubation synergistically accelerates fibrillization of both proteins and drives the formation of pathological inclusions in vitro and in vivo [125]. Similarly, Williams et al. showed in an in vivo mice model that α-Syn fibrils can efficiently cross-seed human tau in a tauopathy and enhance the neuroanatomic spread of seeded tau pathology. Overall, tau–α-Syn copolymers preferentially exacerbate tau seeding [126]. Consistent with these findings, Clinton et al. reported that co-expression of Aβ, tau, and α-Syn in triple-pathology mice (mutant human *SNCA^A53T^* transgene in 3xTg-AD mice) amplifies each lesion and accelerates cognitive decline, supporting a feed-forward crosstalk in mixed AD–DLB phenotypes [127]. Collectively, these studies indicate that Aβ, tau, and α-Syn engage in synergistic cross-seeding interactions that form a feedforward co-pathology loop, exacerbating protein aggregation driving more severe mixed AD–DLB phenotypes.

### 3.3. Mitochondrial Dysfunction and Oxidative Stress in AD Versus DLB

Mitochondrial dysfunction and oxidative stress play an essential part in the pathology of AD. Mitochondrial impairment, leading to faulty energy metabolism and enhanced oxidative damage, has been observed in AD brains. The levels of the antioxidant enzymes catalase, glutathione peroxidase, and superoxide dismutase are reduced in AD pathogenesis, supporting the “mitochondrial cascade” hypothesis [91,128]. Aβ aggregates can trigger a vicious cycle of mitochondrial damage, which further promotes Aβ aggregation [129]. Tau pathology can both trigger and also be driven by mitochondrial bioenergetic failure, impaired mitophagy, and redox imbalance that worsen synaptic health [130].

In DLB, intracellular deposition of the α-Syn inclusions results in mitochondrial dysfunction and oxidative stress [131,132,133]. Studies using Mendelian randomization to probe mitochondrial proteins in DLB has identified several candidates with putative causal links. Higher levels of AIF1 and the ES1 protein homologue were associated with greater DLB risk, whereas higher GLRX2, C1QBP, and mitochondrial GC2 levels were linked to reduced risks [134]. Recent research by Millot et al. has demonstrated that peripheral blood mononuclear cells from patients with AD and DLB show disrupted mitochondrial dynamics, with altered levels of key fusion (MFN2 and OPA1) and fission (FIS1) proteins. These findings underscore that mitochondrial dysfunction, manifesting as an imbalance in the fusion–fission machinery, may be a common pathophysiological signature in DLB as well as in AD; however, further studies are needed to confirm these findings [135].

### 3.4. Neuroinflammation and Cerebrovascular/Endothelial Dysfunction in AD and DLB

Activation of various immune cells, including microglia and astrocytes, in AD patients’ brains is observed as a robust neuroinflammatory response [136,137]. Aβ and pathological tau bind microglial receptors (TREM2 and CD33), transforming homeostatic glia into reactive states that cluster around plaques and tangles [138]. Activated microglia release pro-inflammatory cytokines, chemokines, and inflammasome products such as IL-1, IL-6, TNF-α, and IFN-γ, as well as reactive oxygen species, which exacerbate neurodegeneration [137,139,140]. Microglial cells track Aβ and p-tau; for example, release of cardiolipin from microglia promotes internalization of Aβ [141]. Other brain cells as well as peripheral cells like reactive astrocytes, oligodendrocytes, lymphocytes (adaptive immune system), and peripheral myeloid cells (neutrophils and monocytes) also contribute to pathogenesis and drive neuroinflammation in AD [139].

Cerebrovascular and endothelial dysfunction is another convergent pathological contributor in AD. Blood–brain barrier (BBB) breakdown and endothelial injury causing impaired Aβ clearance may occur at early stage of the disease even before the appearance of cognitive symptoms [142,143,144]. BBB dysfunction leads to the chronic hypoperfusion and cerebral hypoxia, impairing Aβ clearance further enhancing its accumulation and aggregation [145]. Deposition of Aβ in vessel walls (cerebral amyloid angiopathy) impairs vascular integrity and triggers local inflammation [143,146]. A dysfunctional BBB also permits peripheral immune cells to infiltrate the brain; infiltrating leukocytes interact with the neurovascular unit and intensify neuroinflammation and neuronal injury [147,148]. Additionally, vascular risk factors such as hypertension, diabetes, and atherosclerosis favour AD pathology [149].

Evidence from numerous studies in DLB and PDD links neuroinflammation to region-specific changes in microglial state, including activation, in the substantia nigra, putamen, and several cortical regions in early DLB. Whether this response is overall protective or detrimental remains unclear [150].

Intriguingly, a post-mortem cohort study of DLB patients suggests that cortical microglial activation is not a dominant feature. Several inflammatory markers, such as Iba1, HLA-DR, CD68, CD64, CD32b, IL4R, and CHI3L1, were largely unchanged, with lower CD32a, higher CD16, and no increase in neuropil degeneration [77]. Taken together, multiple studies suggest that neuroinflammation in DLB may be time- and region-dependent, whereas AD shows a pronounced, phagocytic microglial phenotype [151]. This evidence is also crucial in shaping how researchers design anti-inflammatory strategies for the two disorders.

Utilizing the autopsy data from the Institute of Clinical Neurobiology, Vienna, Jellinger compared 96 DLB brains (subtyped as “pure” DLB and Lewy-body variant of AD (LBV)), 291 PD brains, and 390 age-matched controls, using routine histology and immunohistochemistry to grade cerebrovascular lesions and cerebral amyloid angiopathy (CAA). This research reported that the cerebrovascular lesion rates in the DLB brains were similar to those of controls and PD, but severe lesions were far less frequent (~2% vs. ~11% in PD and ~6% in controls) and no acute ischaemic or haemorrhagic strokes were present. CAA was common mainly in the LBV brains (~78% vs. ~28% in pure DLB). In DLB, cognitive impairment tracked with neuritic AD co-pathology rather than vascular burden, suggesting a limited cerebrovascular endothelial contribution to DLB dementia [152].

### 3.5. Emerging Pathways from Multi-Omics of AD and DLB

Recent state-of-the-art research such as large transcriptomic meta-analyses and single-cell RNA sequencing is revealing new molecular signatures of AD and DLB. Recently, Johnson et al., using Tandem Mass Tag mass spectrometry (TMT-MS) on large post-mortem AD cohorts, built a proteomic co-expression network and identified enrichment in MAPK signalling and metabolism, which showed strong correlations with AD neuropathology and longitudinal cognitive decline [153]. Using state-of-the-art single-nucleus RNA-seq of human AD prefrontal cortex, Mathys and colleagues identified disease-associated subpopulations and the myelination regulator LINGO1 was consistently perturbed across neuronal and glial populations [154].

Similarly, mouse models revealed that are coordinated shifts across the cortex, hippocampus, and plasma, highlighting altered glucose metabolism (elevated lactate and pyruvate and decreased plasma glucose-6-phosphate) and oxidative-stress signatures, including a significant decrease in cortical galactitol that may reflect its oxidation to an aldehyde and hydrogen peroxide [155]. Similarly, in a complementary human AD Neuroimaging Initiative (ADNI) cohort, untargeted hydrophilic metabolomics and targeted lipidomics identified palmitoleamide, oleamide, diacylglycerols, and ether/plasmalogen lipids as significantly altered at baseline, with several of these (e.g., oleamide) associated with faster progression from mild cognitive impairment to AD [156].

Rajkumar et al. performed post-mortem RNA-seq of the anterior cingulate and dorsolateral prefrontal cortex and identified and validated twelve significant differentially expressed genes at the genome-wide level in DLB: RBM3, ALPI, OXTR, SELE, CSF3, GALNT6, SLC4A1, ABCA13, MPO, SST, RAB44, and CTSG [151]. Greally et al. performed proteomic profiling on pathologically confirmed DLB brains using sarkosyl-insoluble cortical tissue in two subgroups: Tau^positive^ and Tau^negative^ for tau pathology. They found that DLB with Tau^positive^ showed higher level of insoluble tau as well as enrichment of other pathways such as ubiquitin/p62 pathway, vesicle-mediated transport signatures, and Aβ. In contrast, DLB with Tau^negative^ showed increased in cytokine signalling and metabolic pathways and increased abundance of immunoproteasome components (e.g., PSMB8/10 and PSME2), indicating molecularly distinct DLB subtypes [157].

Goralski and co-authors used GeoMx spatial transcriptomics on the human cingulate cortex in disease brains, as well as in a pre-formed fibril (PFF) mouse model, to profile NeuN^+^ neurons with versus without pSer-129–α-Syn (α-Syn phosphorylated at serine 129)-positive inclusions. Inclusion^+^ neurons showed downregulation of synaptic, mitochondrial, proteasome, endolysosomal, and cytoskeletal genes, and upregulation of DNA-damage/repair and complement/cytokine pathways [158]. Using a high-throughput proteomic approach, label-free liquid chromatography–tandem mass spectrometry of cerebrospinal fluid (CSF) samples from DLB patients identified PCSK1N, NPTXR, VGF, PDYN, SCG2, and NPTX2. A three-marker panel (SCG2 + PDYN + VGF) distinguished DLB from non-DLB patients with high accuracy and high specificity, further supporting synaptic dysfunction as a core fluid biomarker signature [158].

In a targeted plasma metabolome profiling of patients with DLB and AD, Pan et al. reported shared reductions in serotonin/taurine and multiple glycerophospholipids/triglycerides, but DLB uniquely showed higher glutamine and lower hydroxylated to nonhydroxylated sphingomyelin ratios. A simple glutamine to lysophosphatidylcholine C24:0 (a common lipid with a 24-carbon chain) ratio distinguished DLB from AD with very high sensitivity and specificity, demonstrating that this ratio could be a potentially crucial biomarker to differentiate these clinically overlapping dementias in diagnostic practice and precision medicine. The main limitations of this pilot study are its small sample size and the absence of replication. If validated in a larger cohort, this approach could provide scalable and cost-effective biomarkers that can be translated to the clinic [159].

Comparative brain proteomics identified what AD and DLB share versus their distinct pathways. For instance, elevated levels of synaptic/mitochondrial proteins are associated with better cognition; however, increased extracellular matrix/complement and autophagy pathway activity is associated with greater cognitive impairment. More specifically, TRIM33 and SLC7A11 were significantly increased, and the autophagy protein p62/SQSTM1 was significantly reduced in DLB compared with AD [160]. Multi-omics, single-cell RNA-seq, bulk proteomics and transcriptomics, single-nucleus transcriptomics, spatial transcriptomics and metabolomics, combined with machine learning and artificial intelligence to analyze complex datasets, are revealing dysregulated genes and pathways and cell-type programmes and prioritizing causal genes at GWAS risk loci, providing a foundation for integrative, data-driven biomarker discovery for AD and DLB [161,162,163].

## 4. Emerging Next-Generation Biomarkers

The current well-established tools for diagnosing AD are CSF biomarkers and neuroimaging. Imaging techniques include advanced PET, such as Aβ-PET (used to visualize Aβ deposition) and tau-PET (used to assess tau pathology), which is used together with ^18^F-fluorodeoxyglucose (^18^F-FDG) PET and structural magnetic resonance imaging (MRI). The latter two serve as indicators of neurodegeneration [164,165]. Major reliable CSF and advanced blood-based biomarkers for pathological changes in AD are Aβ42 (often expressed as the Aβ42/Aβ40 ratio), total tau, phosphorylated tau, neurofilament light chain (NfL), the microRNA subtypes miR-21-5p and miR-451a, and glial fibrillary acidic protein (GFAP) [166,167].

Beyond Aβ and tau, other non-traditional, minimally invasive alternative biomarkers (such as salivary biomarkers (e.g., lactoferrin and acetylcholinesterase), urinary biomarkers (AD7c neuronal thread protein and extracellular vesicle proteins), lipidomic shifts (ceramide/sphingomyelin), synaptic/axonal proteins (neurogranin), circulating microRNAs, and gut/fecal-derived readouts that reflect inflammation, synaptic injury, mitochondrial stress, and membrane lipid dysregulation) can be used to enhance diagnostic accuracy [168] and their use signals a shift to multi-dimensional biomarker assessments. Polykretis et al. demonstrated that atomic force microscopy (AFM) can directly visualize fibrillar structures in crude CSF from patients with AD and other neurological conditions and proposed these features as candidate biomarkers of blood–brain barrier injury and disease progression. However, pre-analytical/analytical harmonization and throughput remain barriers to clinical adoption [169].

Interestingly, detection of spatial navigation ability using the Sea Hero Quest smartphone game is emerging as a sensitive, early “cognitive fingerprint” of AD risk. In large, real-world large demographic samples, navigation performance can flag preclinical risk and has been reported to outperform some standard memory tests, positioning it as an early digital biomarker [41,170].

The well-known current clinical practices for diagnosing DLB are (i) ^123^I-FP-CIT dopamine transporter SPECT (DAT-SPECT) for nigrostriatal uptake, (ii) polysomnography for REM sleep without atonia, and (iii) ^123^I-MIBG cardiac scintigraphy showing reduced cardiac uptake. DAT SPECT offers high specificity; however, it has only moderate sensitivity in prodromal stages [171]. A new line of research has offered a method for detection of α-Syn in biological samples as an additional avenue for biomarker development [172]. α-Syn seed amplification assays (SAAs) such as RTQuIC (Real-Time Quaking-Induced Conversion) and PMCA (Protein Misfolding Cyclic Amplification) show high sensitivity and specificity for detecting PD in CSF samples and hold promise for stratifying synucleinopathies, including DLB, using seeded aggregation kinetics [173]. No blood test has been clinically validated for DLB, although plasma/exosomal α-Syn are under study and some non-traditional matrices, including SAAs for peripheral tissues and body fluid olfactory mucosa and urine, have shown poor agreement with CSF results [174].

Sjaelland and colleagues performed a systematic review that screened 4295 records and included 20 studies and reported that 17 different portable and wearable digital health technologies have been used to capture digital biomarkers of non-cognitive symptoms in DLB, but validation and feasibility reporting remain uncertain. Future digital biomarker–related studies with standardized evaluation are needed before adoption for clinical use [175].

A multiplex approach that integrates core clinical features with neuroimaging and fluid biomarkers is needed to improve the diagnostic accuracy of DLB [176,177]. Beyond diagnostic A/T(N) panels and α-Syn SAAs, prognosis may be informed by nanoscale, label-free readouts from biofluids. Marcuello et al. reviewed AFM platforms that capture the morphology, mechanics, and chemistry of protein assemblies at the single-particle resolution, highlighting their potential to quantify pathological aggregates [178]. If standardized and longitudinally validated (e.g., particle counts, length distributions, and nanomechanical signatures), such AFM-based metrics could complement conventional Aβ/tau/α-Syn measures for risk stratification and pharmacodynamic monitoring, including in DLB. Given the complex nature of AD and DLB pathobiology, no single marker can fully capture disease progression across all stages. Combined use of fluid biomarkers with imaging biomarkers and emerging new biomarkers can provide comprehensive biological insights. Using fluid and imaging measures together provides multilevel insights that better support precision diagnosis and prognosis and treatment monitoring, thereby offering new opportunities for earlier diagnosis and more effective disease management.

## 5. Conclusions and Therapeutic Future Directions

The increasing number of cases of AD and DLB impose steep and rising societal and economic burdens such as caregiver stress and health-system costs. Despite impressive progress in the field, disease-modifying options remain limited aside from recently approved anti-amyloid monoclonal antibodies for AD [179], so there is an urgent need for therapies that halt progression. AD and DLB frequently co-occur and share pathology, complicating diagnosis, prognosis, and medication choice. Hence, it is important to investigate the contribution of co-pathology in each patient. Determining whether a patient has pure AD, pure DLB, or a mixed phenotype is crucial for clinical trial selection and interpretation.

While established imaging and CSF tests are valuable, the current biomarker toolkit for distinguishing mixed versus pure disease is still incomplete. Larger, longitudinal, and standardized studies involving α-Syn SAAs, A/T(N) panels, proteomic/metabolomic signatures, peripheral or blood-based signatures, and rigorously validated digital biomarkers could help in clarifying these complex questions. Leveraging patient-derived induced pluripotent stem cells (iPSCs) and advanced brain organoid models that recapitulate α-Syn, tau, and Aβ pathologies will accelerate target discovery and mechanism-driven pharmacology. High-throughput CRISPR perturbation platforms such as unbiased screens [180] should be used to reveal druggable targets. Modern drug-design toolkits, such as computer-aided drug design, integrative drug design approaches [181], enhanced targeted protein degradation (e.g., PROTACs), molecular chaperone modulators, and peptide/binder design platforms such as BindCraft [182], can be deployed to design and identify selective modulators of α-Syn, tau, and their upstream trafficking/clearance machinery. Future studies should be directed at investigating a combinatorial treatment approach, such as pairing an anti-amyloid agent with an anti-α-Syn therapy, with or without an anti-tau component and other additional strategies for synaptic repair, mitochondrial support, glial-immune modulation, and neurovascular protection.

In conclusion, deeper investigation of the molecular mechanisms, particularly the synergistic interplay between Aβ, α-Syn, and tau, is needed to uncover the mechanisms underlying the overlapping pathology and points of divergence. Clarifying these networks will open up new avenues for earlier and more accurate diagnostics and for rational, mechanism-based therapeutic interventions.

## Data Availability

No new data were created or analyzed in this study. Data sharing is not applicable to this article.

## Abbreviations

The following abbreviations are used in this manuscript:

18F-FDG	18F-fluorodeoxyglucose
A/T(N)	amyloid/tau/neurodegeneration
Aβ	amyloid-β
AD	Alzheimer’s disease
ADNI	Alzheimer’s Disease Neuroimaging Initiative
AFM	atomic force microscopy
BBB	blood–brain barrier
BACE1	β-site APP cleaving enzyme 1
CAA	cerebral amyloid angiopathy
CSF	cerebrospinal fluid
CTF99	C-terminal fragment 99 (of APP)
DALY	disability-adjusted life year
DAT-SPECT	dopamine transporter single-photon emission computed tomography
DLB	dementia with Lewy bodies
GFAP	glial fibrillary acidic protein
GWAS	genome-wide association studies
iPSC	induced pluripotent stem cells
LBD	Lewy body disease
LBV	Lewy body variant (of Alzheimer’s disease)
LMTK2	Lemur Tyrosine Kinase 2
MAPK	mitogen-activated protein kinase
MAPT	microtubule-associated protein tau
MIBG	metaiodobenzylguanidine
miR	microRNA
MRI	magnetic resonance imaging
MSA	multiple system atrophy
NfL	neurofilament light chain
NIA–AA	National Institute on Aging–Alzheimer’s Association
NINCDS–ADRDA	National Institute of Neurological and Communicative Disorders and Stroke–Alzheimer’s Disease and Related Disorders Association
PD	Parkinson’s disease
PDD	Parkinson’s disease dementia
PET	positron emission tomography
PFF	pre-formed fibril
PheWASs	phenome-wide association studies
PMCA	Protein Misfolding Cyclic Amplification
RBD	REM sleep behaviour disorder
REM	rapid eye movement
RNA-seq	RNA sequencing
RT-QuIC	real-time quaking-induced conversion
SAA(s)	seed amplification assays
SPECT	single-photon emission computed tomography
TDP-43	TAR DNA-binding protein 43
TMT-MS	Tandem Mass Tag mass spectrometry
α-Syn	alpha-synuclein
